# Comprehensive predictive modeling in subarachnoid hemorrhage: integrating radiomics and clinical variables

**DOI:** 10.1007/s10143-025-03679-8

**Published:** 2025-06-24

**Authors:** Gemma Urbanos, Ana M. Castaño-León, Mónica Maldonado-Luna, Elena Salvador, Ana Ramos, Carmen Lechuga, César Sanz, Eduardo Juárez, Alfonso Lagares

**Affiliations:** 1 Research Center on Software Technologies and Multimedia Systems for Sustainability (CITSEM), Campus Sur Universidad Politécnica de Madrid (UPM), Madrid, 28031 Spain; 2 Servicio de Neurocirugía, Hospital Universitario 12 de Octubre, Facultad de Medicina, Departamento de Cirugía, Universidad Complutense de Madrid, Instituto de Investigación Sanitaria Hospital 12 de Octubre (Imas12), Madrid, Spain; 3 Servicio de Radiodiagnóstico, Hospital Universitario 12 de Octubre, Madrid, Spain

**Keywords:** Radiomics, Machine learning, Subarachnoid hemorrhage, Glasgow outcome scale, Hydrocephalus, Vasospasm

## Abstract

Subarachnoid hemorrhage (SAH) is a severe condition with high morbidity and long-term neurological consequences. Radiomics, by extracting quantitative features from Computed Tomograhpy (CT) scans, may reveal imaging biomarkers predictive of outcomes. This study evaluates the predictive value of radiomics in SAH for multiple outcomes and compares its performance to models based on clinical data.Radiomic features were extracted from admission CTs using segmentations of brain tissue (white and gray matter) and hemorrhage. Machine learning models with cross-validation were trained using clinical data, radiomics, or both, to predict 6-month mortality, Glasgow Outcome Scale (GOS), vasospasm, and long-term hydrocephalus. SHapley Additive exPlanations (SHAP) analysis was used to interpret feature contributions.The training dataset included 403 aneurysmal SAH patients; GOS predictions used all patients, while vasospasm and hydrocephalus predictions excluded those with incomplete data or early death, leaving 328 and 332 patients, respectively. Radiomics and clinical models demonstrated comparable performance, achieving in validation set AUCs more than 85% for six-month mortality and clinical outcome, and 75% and 86% for vasospasm and hydrocephalus, respectively. In an independent cohort of 41 patients, the combined models yielded AUCs of 89% for mortality, 87% for clinical outcome, 66% for vasospasm, and 72% for hydrocephalus. SHAP analysis highlighted significant contributions of radiomic features from brain tissue and hemorrhage segmentation, alongside key clinical variables, in predicting SAH outcomes.This study underscores the potential of radiomics-based approaches for SAH outcome prediction, demonstrating predictive power comparable to traditional clinical models and enhancing understanding of SAH-related complications.

**Clinical trial number** Not applicable.

**Supplementary Information:**

The online version contains supplementary material available at 10.1007/s10143-025-03679-8.

## Introduction

Nontraumatic subarachnoid hemorrhage (SAH) is a hemorrhagic stroke caused primarily by the rupture of an intracranial aneurysm, associated with high early mortality and complications such as vasospasm, hydrocephalus, and rebleeding [1, 2]. Cranial Computerized Tomography (CT) scans are the diagnostic cornerstone and provide essential information for early risk stratification.

Grading systems such as the Glasgow Coma Scale (GCS) [3]the World Federation of Neurosurgical Societies (WFNS) [4] scale and the Fisher scale [5] are widely used to assess severity and predict outcomes in aneurysmal SAH (aSAH). The modified Fisher scale incorporates intraventricular hemorrhage, improving the prediction of vasospasm [6]. More recently, combined models that integrate clinical status and CT findings, such as the ictWFNS, have shown good prognostic performance and enhanced early risk stratification [7]. However, these approaches rely on visual assessment and predefined qualitative thresholds, which may not capture the full complexity of the pathophysiological process or subtle image-based biomarkers.

Radiomics refers to extracting quantitative data from medical images, revealing hidden information that enhances diagnosis, prognosis, and treatment planning. These features include shape, texture, intensity, and spatial relationships of pixels or voxels within the images. Radiomics has shown promise in oncology, neurology, and cardiology [8, 9].

This study assesses the prognostic value of combining radiomic features and clinical variables to predict key outcomes in aSAH patients. Machine learning models were used to predict mortality, clinical outcome (GOS) [10, 11]vasospasm, and hydrocephalus, based on features extracted from bleeding regions and brain parenchyma in initial CT scans.

Given the heterogeneity of aSAH outcomes, different predictors may be relevant depending on the endpoint. Clinical outcome and mortality are often influenced by hemorrhagic burden and ischemic parenchymal injury [12]while vasospasm is more closely related to cisternal blood, as captured by the modified Fisher scale [13, 14]. Hydrocephalus is typically linked to intraventricular hemorrhage and impaired CSF circulation [15]. These considerations motivated the inclusion of both bleeding and parenchymal segmentations in our models. We further evaluate model performance across input configurations and assess interpretability.

## Materials and methods

**Fig. 1:**
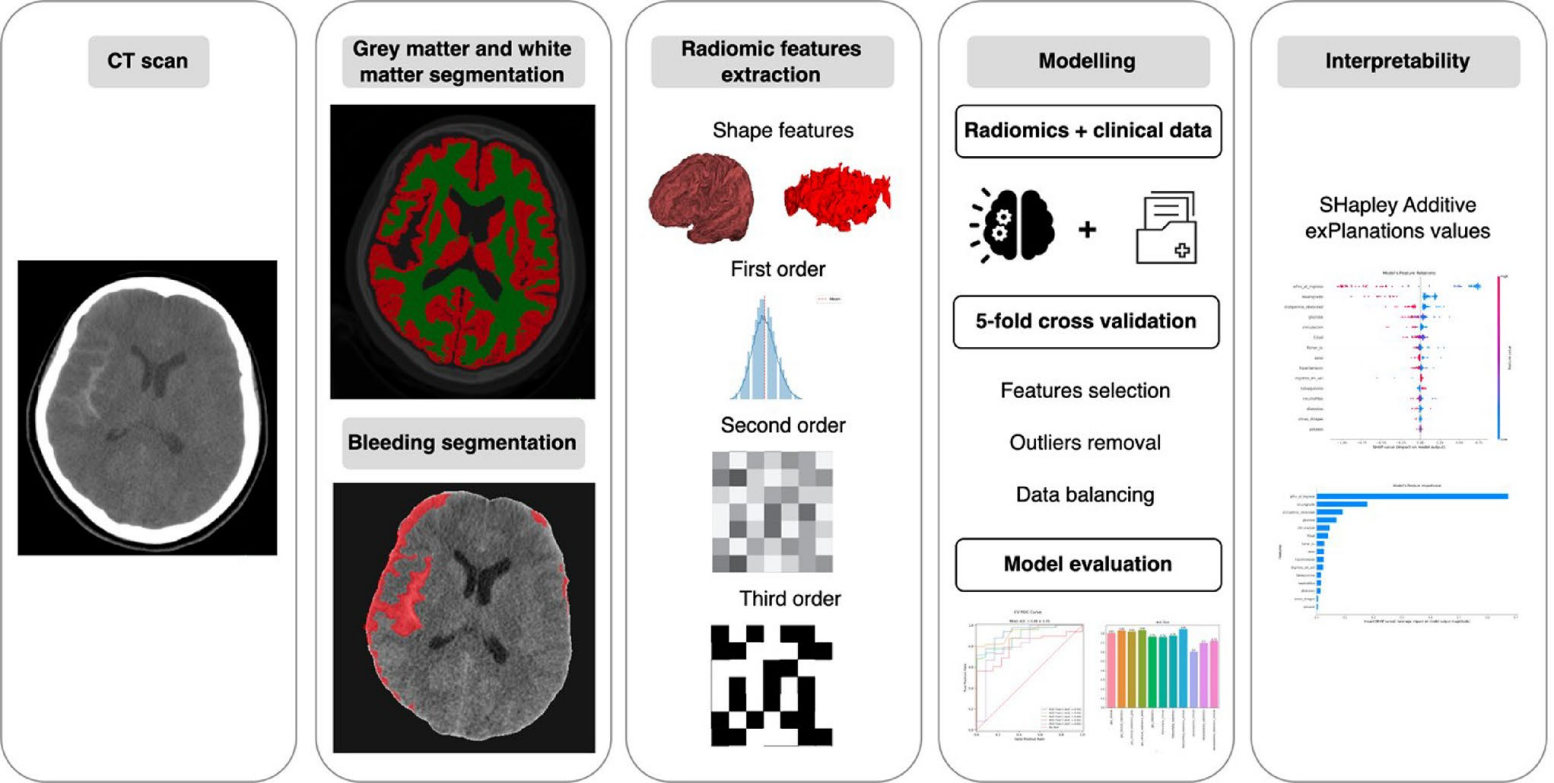
Methodology workflow: Starting from a CT scan, gray matter, white matter, and lesions are segmented. radiomic features are extracted and combined with clinical data for modeling using 5-fold cross-validation. Finally, model interpretability highlights key features and performance metrics

This section describes the dataset analysis (clinical variables and CT images), ROI segmentation (white matter, gray matter, bleeding), radiomic feature extraction, and the methodology for model development, evaluation, and interpretation, as shown in Fig. 1.

### Patient cohort and inclusion criteria

A retrospective dataset of 403 patients was collected from Hospital 12 de Octubre, a single tertiary care center in Madrid, Spain, spanning the period from 2007 to 2023, and was used for model development. Subsequently, an independent dataset comprising 41 patients from 2023 to 2024 was used to evaluate model performance on a more recent cohort, simulating a temporal validation scenario. Inclusion criteria were:

- Diagnosis of spontaneous aSAH confirmed by non-contrast head CT.
- Identification of the causative aneurysm via Computed Tomography Angiography (CTA) and/or Digital Subtraction Angiography (DSA) during hospitalization [17].
- Availability of baseline CT images and complete clinical data at admission.

Exclusion criteria included:

- SAH secondary to trauma or non-aneurysmal causes.
- Lack of follow-up at 6 months post-event.

All CT images were acquired at the time of initial diagnosis. Clinical variables were extracted from electronic health records documented at initial presentation. Outcomes, including mortality and shunt requirements, were based on follow-up reports. While assessors were not formally blinded, these outcomes are objective and routinely documented.

All procedures were part of routine clinical care. Given the retrospective design and use of de-identified data, the study was exempt from formal ethical approval, and the requirement for patient consent was waived by the institutional ethics committee. Clinical and imaging data were pseudonymized and uploaded to the QUIBIM Precision^®^ V3.0.3 platform (Quibim, Valencia, Spain), specifically designated for this study.

### Outcome definitions

The primary assessed outcome was the GOS at six months, with two classification tasks:

- Clinical outcome prediction:
  - *Good outcome*: GOS 4–5 (moderate disability or good recovery).
  - *Poor outcome*: GOS 1–3 (severe disability, vegetative state, or death).
- Mortality prediction:
  - *Survived*: GOS ≥ 2.
  - *Death*: GOS = 1.

Two additional binary outcomes were included:

- **Vasospasm** (*yes/no*): Presence of clinical and radiological findings of cerebral vasospasm.
  - Clinical criteria: New focal neurological deficits or decreased consciousness, not attributable to rebleeding, hydrocephalus, or seizures.
  - Radiological criteria: Vessel narrowing observed on CTA and/or DSA and attributed to vasospasm by the neuroradiologist [18].
- **Hydrocephalus** (*yes/no*): Symptomatic ventricular enlargement requiring definitive cerebrospinal fluid shunting, including cases not tolerating external ventricular drainage during hospitalization or within the six-month follow-up.

### Clinical and image data preprocessing and feature engineering

#### Clinical data and preprocessing

The database initially included 48 clinical variables recorded at hospital admission. Variables with more than 15% missing data were excluded from further analysis. This process resulted in a final set of 20 clinical variables summarized in Table 1, and definitions along with diagnostic thresholds for comorbidities are detailed in Supplemental Table 1.

A comparative statistical analysis was performed between the training and validation dataset and the test set. Categorical variables were analyzed with the Chi-squared test, and effect size was measured using Phi for 2 × 2 tables and Cramér’s V for larger tables. Continuous variables were compared with independent t-tests, and Cohen’s d as used for effect size. A significance level of *P* =.05 was applied [19].

Missing data were handled using Multiple Imputation by Chained Eq. [20] (MICE), with linear regression for continuous variables and logistic regression for categorical ones. Five imputations were performed to improve reliability and validity.

#### Image segmentation

**Fig. 2:**
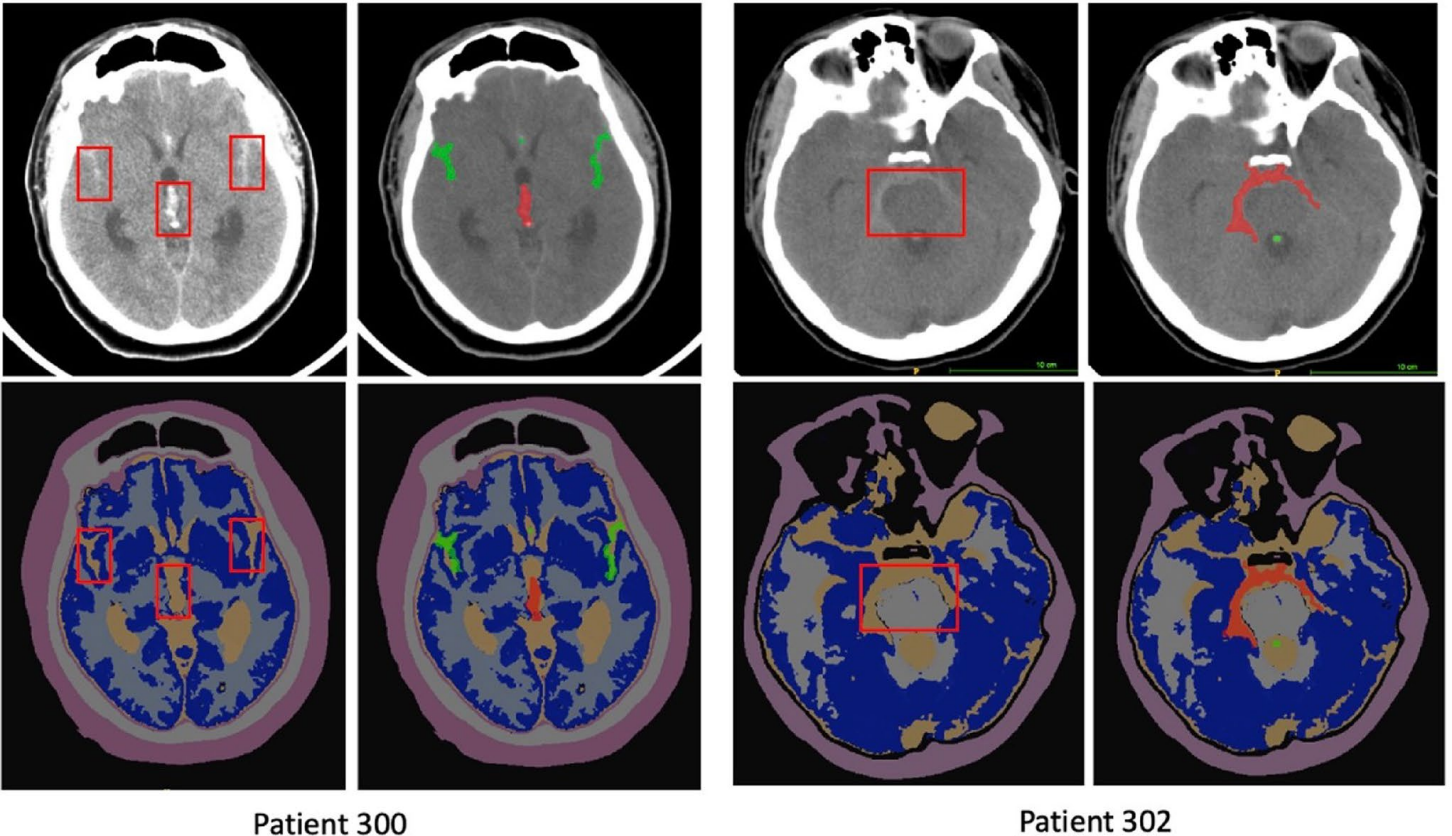
Brain tissue segmentation using CTseg. The right column for each patient shows the original image with the brain segmentation. In the left column, the bleeding segmentation is also represented

Radiomics features were extracted from gray matter, white matter, and bleeding segmentations, as these regions can influence patient outcomes [21–23]. CTSeg, an atlas-based algorithm for brain CT segmentation [24, 25] was used to classify six regions: gray matter, white matter, cerebrospinal fluid, skull, soft tissue, and background. Based on SPM12 tool, CTSeg has been validated in numerous studies [26, 27]. Blood regions are often classified as cerebrospinal fluid, which is unlikely to significantly affect the radiomics from gray and white matter. Figure 2 shows the segmentation of these regions and the HSA, with a bounding box, from two randomly selected cases.

Bleeding segmentation was performed with a pretrained Vision Transformer (ViT) model [28]. Images were preprocessed to exclude the skull, and lesion segmentation was validated against manual semiautomated volumes from 255 patients [12]. Two validation approaches were used for bleeding automated segmentation: first, a clinical database parameter derived from semi-automated manual segmentation [12, 15] and second, a review by an experienced neurosurgeon.

### Image preprocessing

CT images were resampled to 1 × 1 × 1 mm³ isotropic voxels. Intensity values were discretized with a fixed bin width of 25 Hounsfield units to ensure reproducibility across scans. Z-score normalization was applied to all images prior to radiomics feature extraction, standardizing intensity distributions across all patients.

### Radiomics feature extraction

Radiomics features were extracted from brain tissue and bleeding segmentation using PyRadiomics v3.0.1 [29], following IBSI guidelines [30]. A total of 1379 radiomic features were extracted per region: 14 shape, 18 first-order, and 73 s-order features, with an additional 1365 derived from filtered images using five filter types (square, exponential, logarithm, eight-level Haar wavelet, and Laplacian of Gaussian with σ = 0.5, 3.0, and 5.0).

Radiomics from gray and white matter were combined to create a brain tissue signature. Predictive models were developed separately for brain tissue and bleeding radiomics, enabling comparison of their clinical utility.

Models were developed using three feature sets: clinical variables only (20 features), radiomics only, and a combination of both. Radiomics-only models included 1,379 features for bleeding segmentation and 2,758 for gray/white matter segmentation. Combined models integrated clinical variables with 1,399 (bleeding) and 2,778 (gray/white matter) features, respectively.

### Feature engineering

Spearman correlation analysis was performed to identify highly correlated features within both clinical and radiomic datasets. Features with a correlation above 90% were excluded to prevent redundancy. Additionally, feature selection was performed using the Minimum Redundancy Maximum Relevance (MRMR) [31] method within each cross-validation fold, ensuring that only the most informative and non-redundant features were retained for model development.

### Model development and evaluation

Model development followed a 5-fold stratified cross-validation. In each fold, 80% of the data was used for model development, with internal validation for tuning, and 20% as test set. All steps were confined to training data to prevent information leakage.

To clarify the terminology: the **training set** is used for model fitting, including feature selection and hyperparameter tuning; the **validation set** is an internal split within the training data for tuning during cross-validation; the **test set** refers to the held-out fold in 5-fold cross-validation, used to assess performance on unseen data; and the **independent test cohort** is a separate external dataset of 41 patients (2023–2024), excluded from model development, used to evaluate generalizability.

**Fig. 3:**
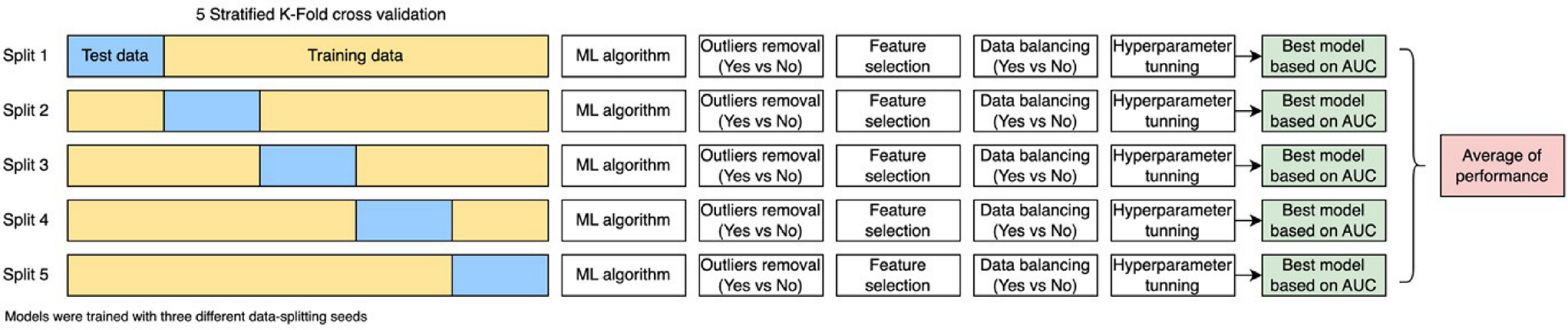
Model development used 5-fold stratified cross-validation with three random seeds. Each split included a unique test (blue) and training set (yellow). Models underwent outlier removal, feature selection, data balancing, and hyperparameter tuning. The best model per fold was selected by AUC, and overall performance was averaged

Figure 3 illustrates the model development methodology. For each fold:

1. **Algorithms: **
2. **Random Forest** [32] (RF) Extra Trees Classifier [33] (ExtraRF) and Extreme Gradient Boosting [34] (XGBoost) were tested.
3. **Feature Selection**: MRMR [31] was used to select 5–20 features for clinical models and 5–30 for radiomics models.
4. **Hyperparameter Tuning**: Grid search [35] optimized hyperparameters using AUC as the metric.
5. **Outlier Removal**: Isolation Forest [36] was tested for detecting and removing outliers.
6. **Data Balancing**: ADASYN [37] was applied to improve class balance, tested with and without usage.

The entire procedure was repeated using three random seeds to assess the consistency and robustness of the results.

The best-performing configuration in each fold was selected based on validation AUC. Performance was reported as the average and 95% confidence intervals across folds and seeds. Additionally, each model obtained during cross-validation (across folds and seeds) was evaluated on the independent test cohort to assess temporal generalizability.

Model predictions were generated using probability outputs from scikit-learn [38] and XGBoost APIs [33]. Besides AUC, metrics such as Balanced Accuracy (BalAcc), Sensitivity (Sens), and Specificity (Spec), F1-Score, Accuracy (Acc) and confusion matrices were monitored [39, 40]. Calibration curves [41] and Brier scores [42] were also computed to evaluate the reliability and accuracy of predicted probabilities.

SHAP (SHapley Additive exPlanations) values are a method for interpreting machine learning models by fairly attributing each feature’s contribution to the model’s predictions, based on Shapley values from cooperative game theory [43].

Furthermore, to enable a robust comparison of model results with established scales in the literature, the ictWFNS [7] score derived from Subarachnoid Hemorrhage Early Brain Edema Score (SEBES) [44]Le Roux [45] and Hijdra [13] scales was computed for the cases in the test set (*n* = 41). Logistic regression models will be fitted to assess the predictive value of ictWFNS for each clinical outcome.

## Results

### Dataset characterisation

**Fig. 4:**
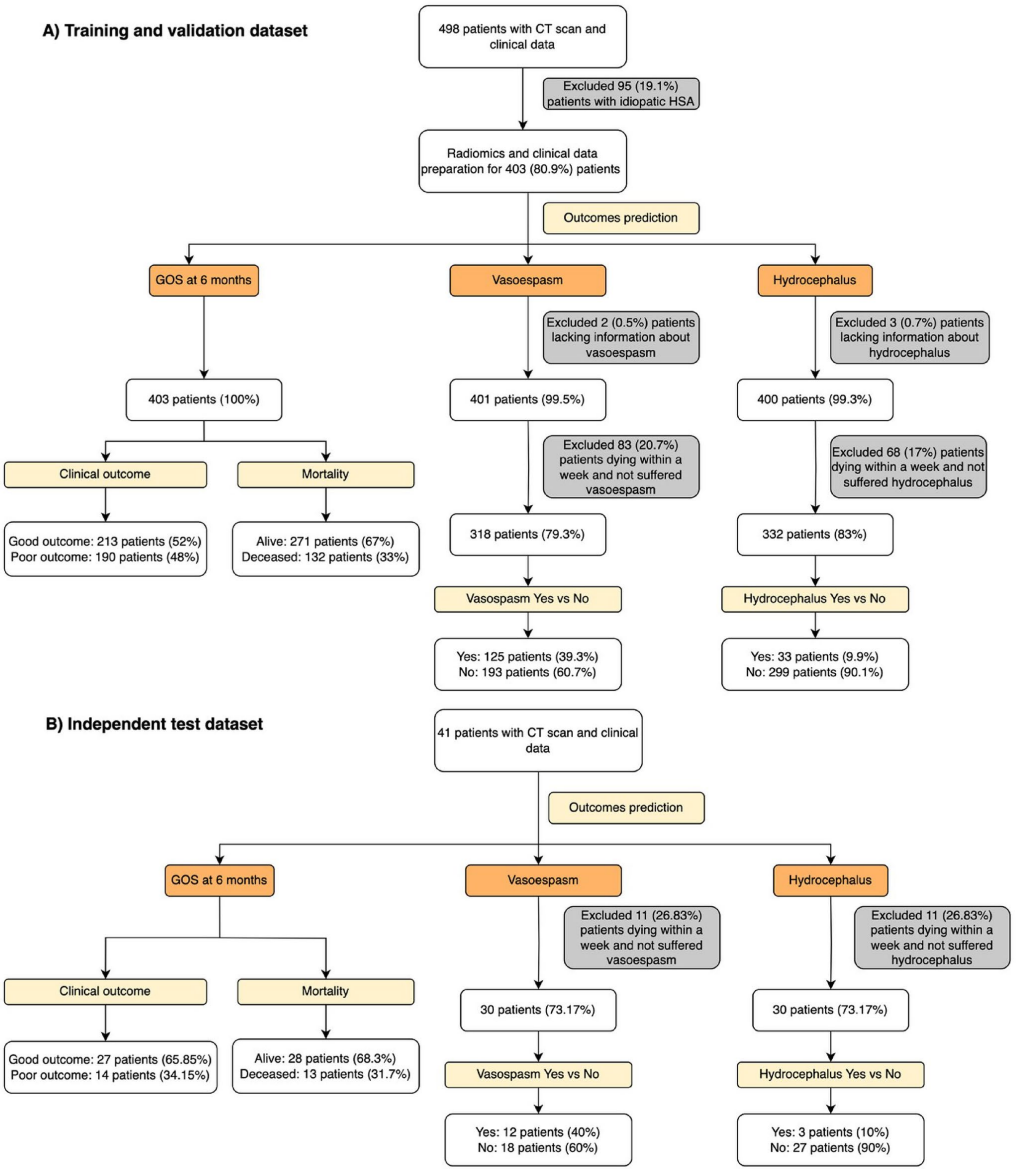
Patient selection flow diagram, indicating the distribution of patients across different outcome categories

From an initial cohort of 498 patients (2007–2023), 403 with confirmed aneurysmal SAH were included. Early deaths and cases with missing outcome data were excluded. As a result, the number of patients varied by predictive model: 194 for poor clinical outcome, 133 for six-month mortality, 125 for vasospasm (96 clinical, 29 radiological), and 33 for long-term hydrocephalus (Fig. 4a).

The training cohort comprised 68.5% females, with a mean age of 52 ± 18 years. Hypertension (41.7%), smoking (28.5%), and diabetes (18.6%) were the most frequent comorbidities. The majority presented with WFNS grade 1 (42.6%) and modified Fisher grade 3 (66.5%). Only admission variables were considered and treatment-related features were excluded to ensure prognostic utility. CT images were acquired primarily using Philips Brilliance 6 (*n* = 380), with a mean in-plane resolution of 0.46 mm (SD 0.05) and slice thickness of 1.97 mm (SD 0.98). Imaging acquisition parameters are summarized in Supplemental Fig. 1.

A distinct temporal test set of 41 patients (2023–2024), acquired mainly with a different CT scanner than the training cohort, was used to assess generalizability (Fig. 4b). This cohort comprised 14 patients with poor outcome, 13 deaths, 13 with radiological vasospasm, and 3 with hydrocephalus. Most CTs were acquired using GE Revolution EVO (*n* = 38), with a mean pixel size of 0.49 mm (SD 0.04) and slice thickness of 1.40 mm (SD 0.88).

The clinical characteristics of the training/validation and test cohorts are summarized in Table 1, along with the results of a statistical comparison. Significant differences were observed between cohorts in the prevalence of hypertension, smoking, and alcoholism, as well as in WFNS and modified Fisher grades at admission. Differences were also found in age, glucose levels, and platelet counts.

**Table 1:** Clinical characteristics of patients in the training/validation and test datasets, with statistical comparison

Variable	Train/Validation	Test	*p*-value	Effect Size	Significant?
Categorical Variables (%)				Phi/ Cramer’s V	
Gender (Female)	68.5%	58.5%	0.05	0.15	No
Hypertension (Yes)	41.7%	51.2%	0.03	0.22	Yes
Smoking (Yes)	28.5%	43.9%	0.01	0.31	Yes
Diabetes (Yes)	18.6%	14.6%	0.33	0.10	No
Dyslipidemia/Obesity (Yes)	17.9%	48.8%	0.00	0.64	Yes
Alcoholism (Yes)	5.7%	14.6%	0.01	0.27	Yes
WFNS score (≥ 4)	38.4%	61.0%	0.00	0.54	Yes
Modified Fisher (≥ 3)	79.4%	90.2%	0.03	0.21	Yes
Posterior circulation	9.0%	0%	0.04	0.24	Yes
**Numerical Variables (Mean ± SD)**				**Cohen’s D**	
Age (years)	52 ± 18	59 ± 14	0.03	-0.43	Yes
Glucose (mg/dL)	151 ± 56	186 ± 63	0.02	-0.58	Yes
Platelets (10⁹/L)	698 ± 101	238 ± 53	0.00	5.69	Yes

### Image segmentation

**Fig. 5:**
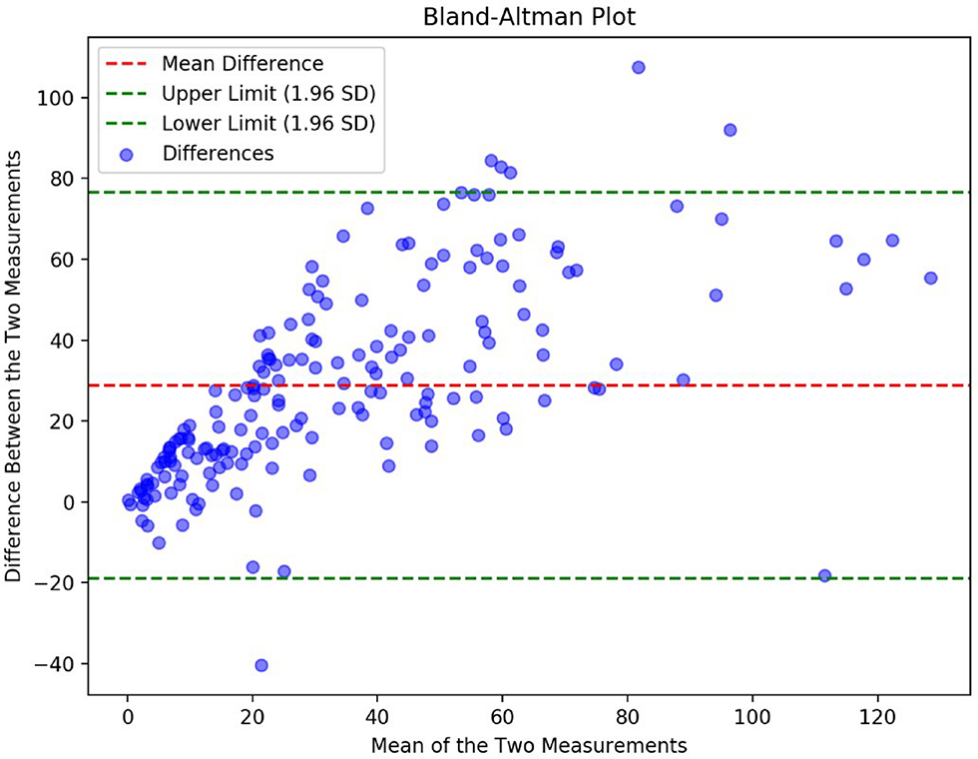
Bland-altman plot comparing bleeding volume (mL) from manual and automatic segmentations. The central line shows the mean bias; outer lines indicate 95% limits of agreement

Validation of the automatic bleeding segmentation was performed by comparing model predictions with clinical database parameters, followed by expert review from an experienced neurosurgeon. For the clinical database, bleeding volumes for 255 patients averaged 20.14 mL (range: 0–120.57 mL). In comparison, the automated model reported an average of 48.95 mL (range: 0–156.09 mL). Bland-Altman plot (Fig. 5) reveals a bias of 25 mL higher for automatic segmentation. Larger discrepancies were noted for higher bleeding volumes, consistent with other studies reporting differences from 15 mL [46] to more than 20 mL [47] particularly for large hemorrhages where algorithms struggle with contour delineation. Points near (0,0) likely reflect cases with small bleeds segmented manually but missed by the automatic model, reflecting its limitations in detecting low-volume hemorrhages.

Additionally, a neurosurgeon visually assessed 20 randomly selected segmentations across varying hemorrhage volumes and locations. Overestimations were mainly observed on convexity surfaces, while intrahemispheric regions were more accurate. Supplemental Table 2 illustrates five representative cases, illustrating typical over- and under-segmentations patterns.

### Performance metrics of the models

**Fig. 6:**
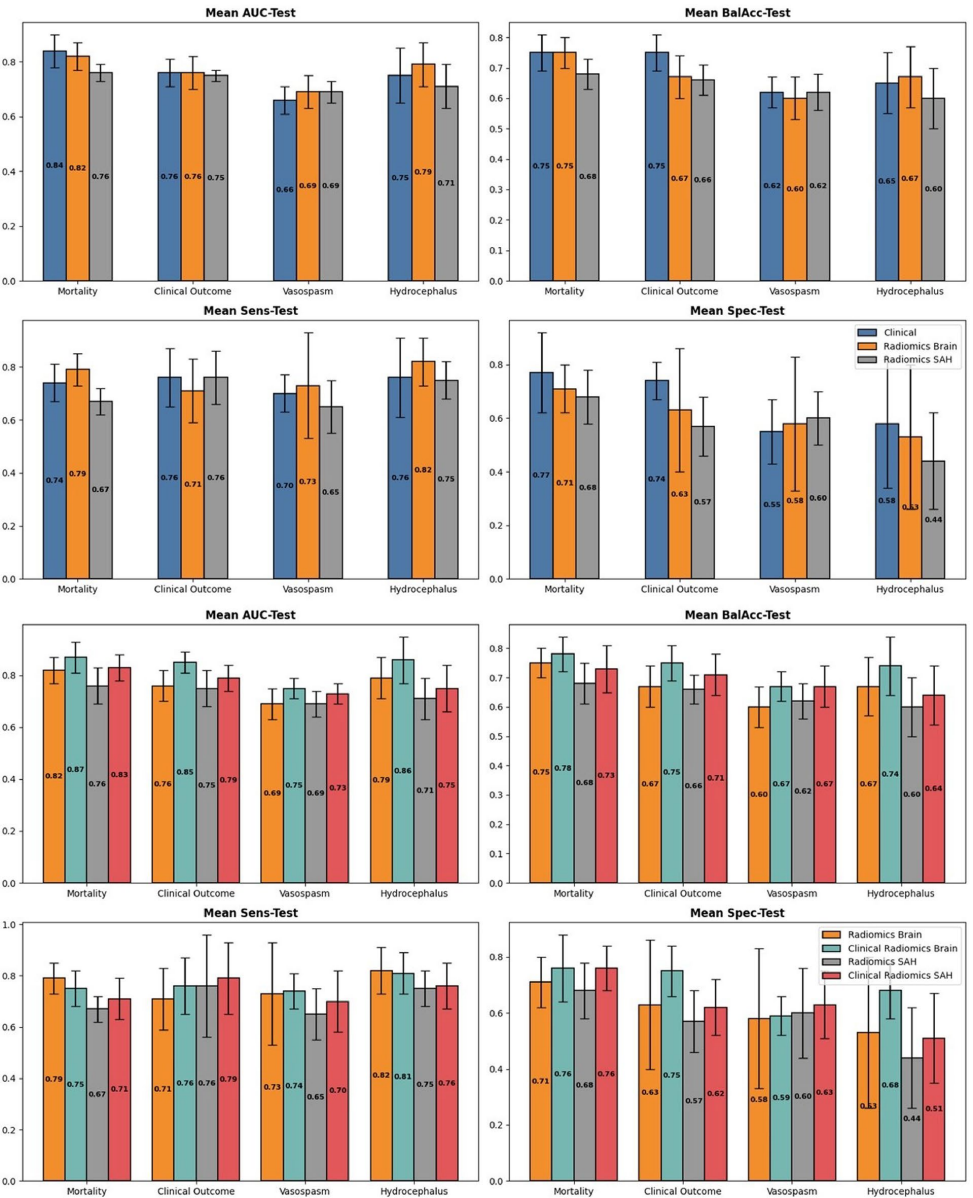
Model performance was evaluated using AUC, balacc, sens, and spec on the test set, with confidence intervals. The upper plot compares model performance with clinical data, radiomics from white/gray matter segmentation, and bleeding region radiomics. The lower plot compares performance using radiomics alone versus combining clinical data with radiomics from both regions

First, the predictive power of radiomics was compared to clinical data, as shown in the upper graph of Fig. 6. The lower graph of Fig. 6 depicts models using only radiomics versus combining radiomics and clinical data. The comparison includes radiomics from bleeding regions and from gray and white matter regions.

Performance metrics (AUC, BalAcc, Sens, and Spec) were used to evaluate each model. For each, 15 variations were generated, with 95% confidence intervals based on predictions from 5-fold cross-validation using 3 different seed partitions.

Figure 6 upper shows that radiomics performs comparably to traditional clinical variables. Radiomics-based models perform comparably to those using clinical variables, with overlapping confidence intervals across outcomes. AUC values range from 0.76 to 0.84 for mortality, 0.75–0.76 for clinical outcome, 0.64–0.69 for vasospasm, and 0.71–0.79 for hydrocephalus. For hydrocephalus in particular, class imbalance warrants emphasis on BalAcc (0.60–0.75), Sens (0.75–0.82), and Spec (0.44–0.58), with larger confidence intervals indicating less reliability.

Figure 6 lower despicts models using only radiomics or combining clinical and radiomic data. In the mortality model, AUC ranges from 0.76 to 0.87, with clinical outcomes from 0.75 to 0.85. Vasospasm AUC ranges from 0.69 to 0.75, and hydrocephalus ranges from 0.71 to 0.86. In hydrocephalus similar trends are observed in BalAcc (0.60–0.74), Sens (0.65–0.81), and Spec (0.44–0.68).

The best-performing configurations generally avoided outlier removal, applied data balancing, and used ExtraRF, RF, or XGBoost algorithms. Radiomics models typically selected 10–30 features, while clinical models used 5–20. Supplemental Fig. 2 shows calibration curves and Brier scores across cross-validation folds for models using clinical data, radiomics, or both.

Supplemental Fig. 3 presents model performance stratified by age (< 70 vs. ≥70 years). Models consistently performed better in younger patients, with radiomics-based models showing smaller AUC differences across age groups. Supplemental Table 3 reports model performance by clinical grade (WFNS 1–3 vs. 4–5). Models tends to classify patients with good grades more accurately.

**Fig. 7:**
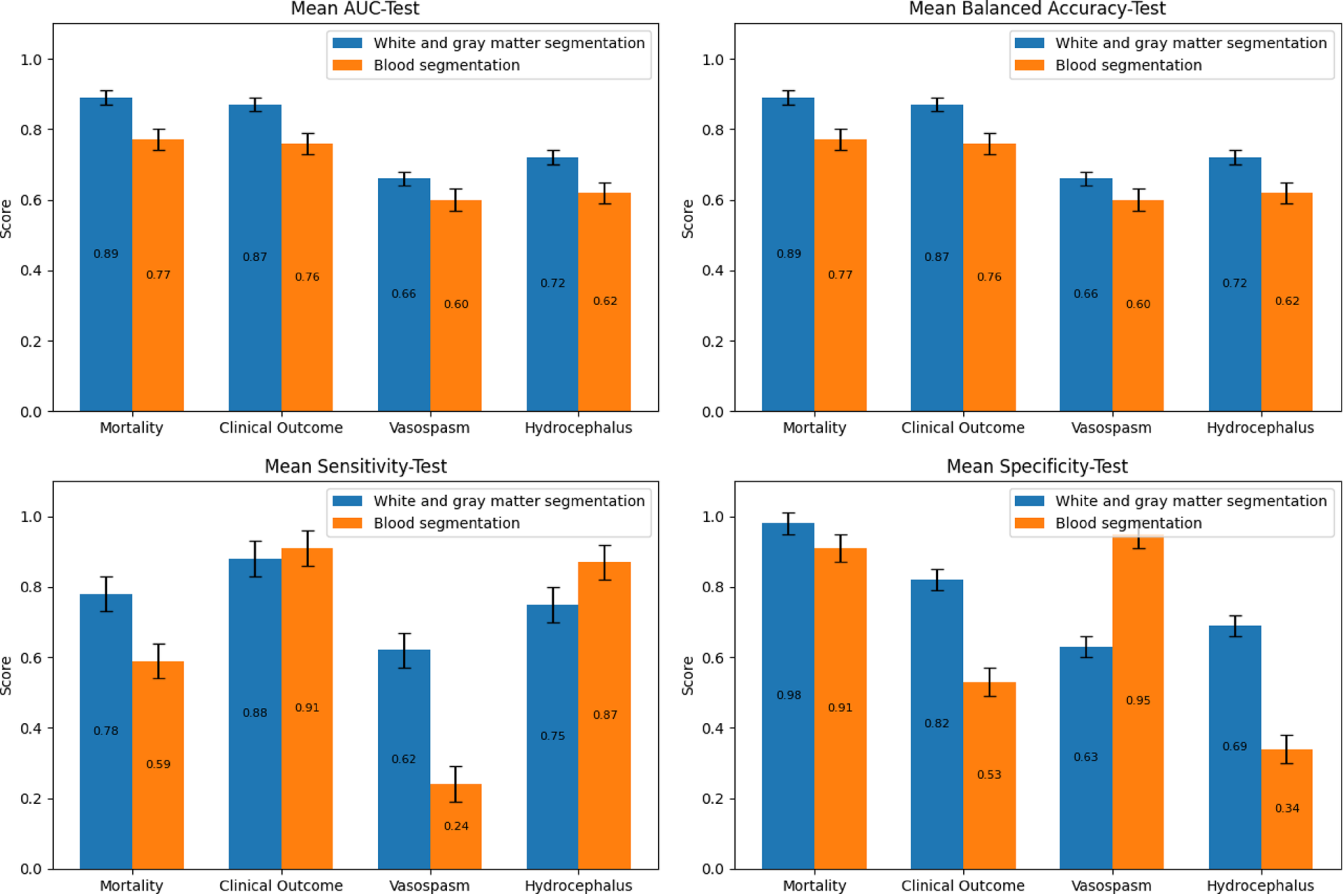
Model performance results on the independent test set. The models combined radiomic and clinical data. Blue bars represent results based on radiomics from gray and white matter, whereas green bars correspond to features extracted from bleeding segmentations

Finally, models combining radiomics and clinical variables were evaluated on the independent test set (Fig. 7), comparing white/gray matter (blue) versus blood segmentation (green). The highest AUC was observed for mortality prediction (0.88 vs. 0.75). For clinical outcome, AUCs were 0.85 vs. 0.72; for vasospasm and hydrocephalus, 0.62 vs. 0.58 and 0.71 vs. 0.60, respectively. Blood based models showed low sensitivity for vasospasm (e.g., 0.21) and poor specificity for hydrocephalus (e.g., 0.33), while white/gray matter-based models yielded more robust and balanced performance across all tasks.

To benchmark predictive performance, a logistic regression using the ictWFNS score (*n* = 41). As shown in Table 2, ictWFNS achieved modest AUCs for mortality (0.70) and poor clinical outcome (0.70), but showed limited predictive value for vasospasm (AUC = 0.60) and hydrocephalus (AUC = 0.56). In contrast, radiomics-based models demonstrated superior performance across all outcomes. Table 2 also reports Sens, Spec and the p-value associated with ictWFNS, which reflects the significance of the score as a predictor in the logistic regression model. To support reproducibility, a Supplemental Table 4 details the distribution of SEBES, Hijdra, and LeRoux scores used to compute the ictWFNS.

**Table 2:** Predictive outcome performance of IctWFNS and brain radiomics-based models in the test cohort (*n* = 41)

Outcome	Model	AUC	Sens.	Spec.	*p*-value ictWFNS*
Mortality	ictWFNS	0.696	0.455	0.947	0.049
	Brain radiomics model	0.89	0.78	0.98	—
Clinical Outcome	ictWFNS	0.699	0.625	0.643	0.044
	Brain radiomics model	0.87	0.88	0.82	—
Vasospasm	ictWFNS	0.596	0.500	0.615	0.243
	Brain radiomics model	0.66	0.66	0.62	—
Hydrocephalus	ictWFNS	0.560	0.000	1.000	0.726
	Brain radiomics model	0.72	0.75	0.69	—

*p-value from logistic regression. Not applicable for non-parametric radiomics models

Finally, Supplemental Figs. 4 and 5 present the F1-score and accuracy metrics, along with confusion matrices for each outcome, separately for the validation and independent test sets.

### Models interpretability

**Fig. 8:**
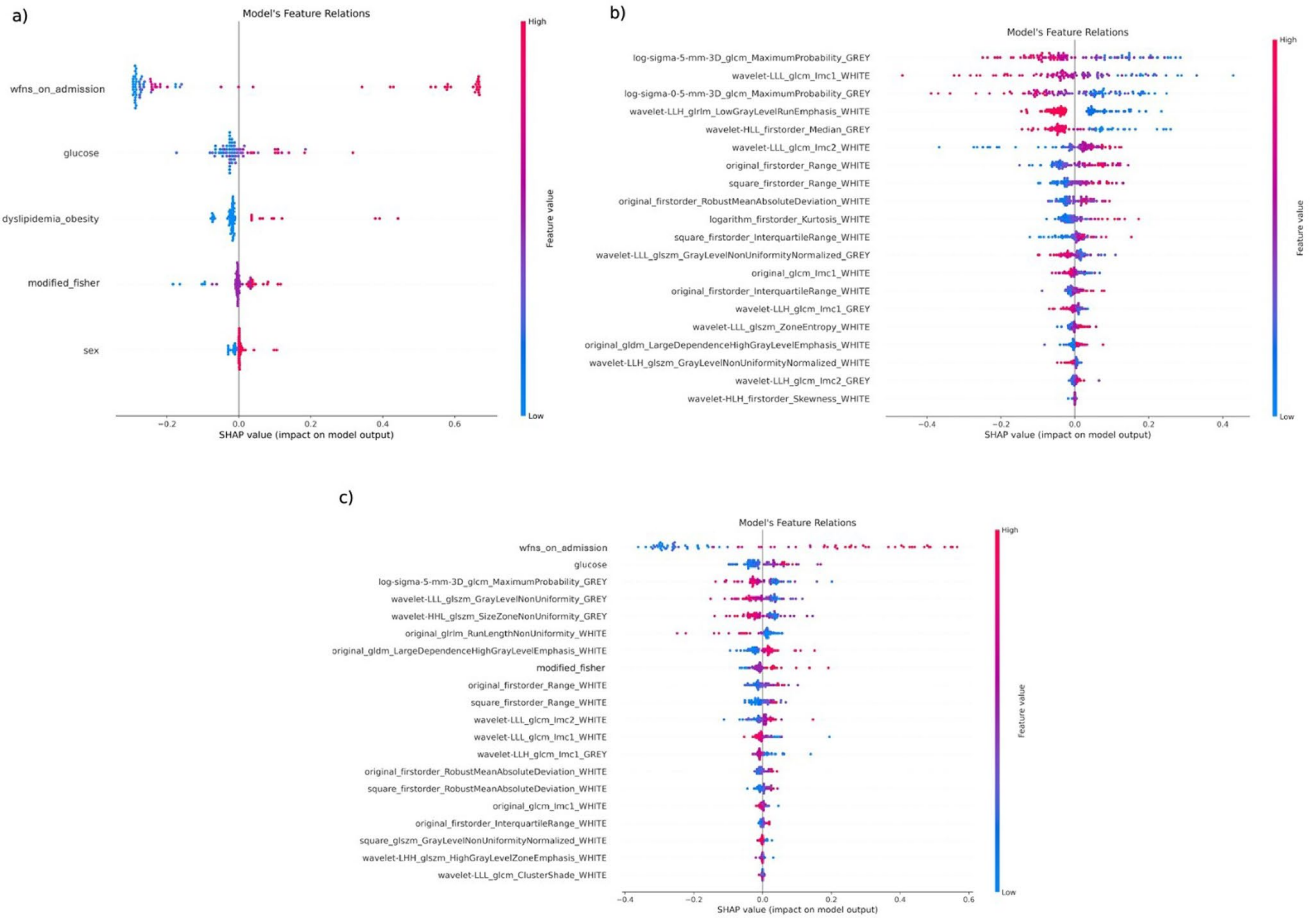
SHAP values diagrams for predicting mortality from white and grey matter segmentation models using (**a**) clinical, (**b**) radiomics and (**c**) combining both as input

Figure 8 shows SHAP value plots for the mortality model based on white and gray matter segmentation, which achieved the highest AUC. Positive values indicate features linked to higher risk, and negative values to better outcomes. Features are ranked by impact, with the most relevant at the top.

Additional SHAP values are provided in Supplemental Fig. 6. In summary, WFNS at admission is the most significant feature across all models. Glucose levels are also important for most outcomes. Radiomics models show that texture-based features in gray and white matter are crucial for predicting clinical outcomes, reflecting structural changes in the brain that are key to determining patient prognosis and risks.

## Discussion

This study assessed the predictive value of radiomics and clinical data across multiple outcomes in patients with aSAH. Radiomic features were extracted using two segmentation strategies: bleeding and parenchymal tissue (gray and white matter). Three types of predictive models were developed and validated: one based on clinical variables, one on radiomics, and one combining both. Machine learning models were trained and validated using cross-validation to ensure robust performance assessment and to evaluate the incremental benefit of integrating radiomics with clinical information.

Overall, models demonstrated reasonable performance, particularly for mortality and clinical outcomes (AUC ~ 85%). Vasospasm prediction was lower (AUC 75%), while hydrocephalus models still performed well despite class imbalance (AUC 86%). Radiomics showed comparable performance to clinical models, with overlapping confidence intervals suggesting they can serve as competitive alternatives to traditional clinical predictors.

Importantly, combined models preserved high performance on the independent test set (AUCs: 89% mortality, 87% clinical outcome), though performance dropped for vasospasm (− 9%) and hydrocephalus (− 12%), possibly reflecting discrepancies in case definitions and variations in clinical variables. Moreover, imaging protocol heterogeneity may have further impacted generalizability.

Given that age is a known prognostic factor in aSAH, patients were stratified using a 70-year cutoff. Notably, better and more consistent AUCs were observed in younger individuals, suggesting greater predictive value of both radiomic and clinical features in this group. This finding may reflect higher brain reserve in younger patients and greater vulnerability to complications in older ones. SEBES [44] also shows age-dependent prognostic value, further supporting age-based stratification.

From a modeling perspective, tree-based algorithms (RF, ExtraTrees, XGBoost) consistently performed well. Clinical models typically required 5–20 features, while radiomics models used 10–30. Data balancing improved performance, whereas outlier removal had minimal impact. However, including too many features increased overfitting risk and reduced generalizability.

Radiomics models based on bleeding segmentation performed similarly to parenchymal models during cross-validation but showed lower performance in the independent test set. Although bleeding segmentation was included for its clinical relevance to vasospasm and hydrocephalus, it often overestimated blood volume, introducing bias and limiting predictive value. In contrast, parenchymal features proved more consistent and predictive, especially for mortality and clinical outcomes. This illustrates a trade-off: bleeding segmentation is faster but less reliable, whereas parenchymal segmentation is more robust but resource-intensive.

Clinical and radiomics models demonstrated similar predictive power both individually and in combination. Radiomics are particularly valuable in scenarios where clinical information is incomplete, missing, or unreliable, such as in emergency settings or retrospective studies. In such cases, radiomics-derived models could serve as a viable alternative for early risk stratification, as they rely solely on routinely acquired CT scans. This expands their applicability and highlights their potential role in automated, reproducible, and scalable decision-support systems.

To benchmark model performance, the ictWFNS [7] score was calculated in the independent test cohort. It showed moderate AUCs (~ 0.70) for mortality and poor outcome but lower predictive value for vasospasm and hydrocephalus. By comparison, radiomics-based models outperformed ictWFNS across all outcomes, particularly for mortality (AUC 0.89) and poor clinical outcome (AUC 0.87). Nevertheless, these results are limited by the small test cohort (*n* = 41), and validation in larger, multicenter populations is warranted. To further contextualize our results, Table 3 compares the performance of our parenchymal radiomics–clinical model with that of previously published models based on traditional grading scales.

**Table 3:** Comparative performance of proposed models and published studies in predicting key outcomes in aSAH, including AUC, sensitivity, and specificity where reported

Outcome	Metric	Radiomics-Based Models	WFNS / Fisher-Based Studies	Reference
Mortality	Sens and Spec	Sens 0.75 Spec 0.76 at 6 months	WFNS Sens 0.89, Spec 0.19 hWFNS Sens 0.44, Spec 0.93 at 6 months	Raabe et al. 2022 [50]
Clinical Outcome	AUC	0.85 At 6 months	WFNS AUC ~ 0.837; modWFNS AUC ~ 0.839; At 3 months	Nguyen et al., 2023; Hofmann et al., 2023 [51]
Vasospasm	AUC	0.75 At 6 months	Fisher/modified Fisher AUC ~ 0.65–0.70 At 3 months	Couret et al., 2024 [52]
Hydrocephalus	AUC	0.86 At 6 months	Intraventricular Hemorraghe score AUC ~ 0.85 at 3 months modified Fisher AUC ~ 0.81 At 1 month	Couret et al., 2024 [52] Rao et al. 2024 [53]

Radiomics models demonstrate moderate to strong predictive performance compared to traditional grading systems like WFNS and Fisher in aSAH. For 6-month mortality, radiomics models yield balanced sensitivity (0.75) and specificity (0.76), whereas WFNS shows high sensitivity (0.89) but low specificity (0.19), and hWFNS improves specificity (0.93) at the expense of sensitivity (0.44) [50]. Thus, radiomics offer more balanced discrimination. For clinical outcome, radiomics outperform WFNS and modWFNS [51]achieving an AUC of 0.85. They also show competitive AUCs for vasospasm (0.75) [52] and hydrocephalus (0.86) [53]. Importantly, radiomics models support longer prediction windows, enabling broader clinical decision-making.

SHAP analysis enhanced model interpretability by highlighting key radiomic features contributing to predictions. Among them, third-order texture descriptors were the most influential, potentially capturing tissue heterogeneity, edema, or complex hemorrhagic patterns [54, 55]. However, these interpretations remain hypothetical and requires further validation.

Clinical features also contributed meaningfully. The WFNS score at admission consistently appeared as the most impactful feature, in line with its well-established prognostic value [3, 56]. Other relevant predictors included modified Fisher grade, glucose levels, age, smoking status, lymphocyte count, and neutrophil count. Their consistent importance across models and alignment with prior studies reinforce the robustness and clinical relevance of our findings [57, 58].

Unlike previous studies focused solely on bleeding-based radiomics [59, 60]this work systematically compared bleeding and parenchymal segmentation. Radiomics from gray and white matter yielded superior and more generalizable performance, underscoring their added value in outcome prediction.

Despite these promising results, this study has limitations. Its retrospective, single-center design may introduce selection bias and limit generalizability. The small sample size and class imbalance reduce statistical power and may affect model calibration, despite the use of oversampling and cross-validation. The high dimensionality of radiomic data increases the risk of overfitting, and although MRMR was used for feature selection, other strategies were not explored.

Moreover, the limited test cohort, particularly in subgroups like hydrocephalus, further constrains performance estimates. Radiomic features are sensitive to acquisition parameters and artifacts; no scanner harmonization was applied, and potential confounders such as dental implants were not evaluated.

The automatic bleeding segmentation consistently overestimated hemorrhage volume by an average of 25 mL compared to reference annotations. In addition, the algorithm appeared to miss or under-segment some smaller hemorrhages, which may have further impacted the reliability of radiomic features. Improving segmentation accuracy via algorithm refinement, alternative models, or manual correction is essential to enhance model reliability.

Treatment variables were not included, and the test set, though temporally independent, came from the same center. Also, outcome definitions, especially for vasospasm, may vary and affect generalizability. Thus, external multicenter validation is needed. Additionally, the analysis did not stratify performance by sociodemographic factors beyond age and sex. Future research should address these gaps, promote protocol harmonization, and prioritize clinical translation.

In conclusion, combining radiomics with clinical data holds promise for real-time risk stratification, personalized follow-up, and early intervention. Prospective validation, harmonization frameworks, and implementation pathways will be essential for successful clinical integration.

## Conclusions

This study demonstrates that radiomics derived from both brain parenchyma and hemorrhage segmentation can predict key outcomes in aSAH with performance comparable to established clinical models. Radiomics- and clinical-based models yielded AUCs exceeding 85% for mortality and poor clinical outcome, while models for vasospasm and hydrocephalus also achieved satisfactory performance despite class imbalance (AUCs of 75% and 86%, respectively).

Gray and white matter segmentation generally provided superior predictive performance compared to bleeding-based approaches, though both were effective. Interpretability analysis identified relevant radiomic and clinical features associated with worse prognosis, consistent with previous evidence.

These findings support the integration of radiomics into prognostic modeling for aSAH. Future work should focus on refining bleeding segmentation accuracy, validating results in external cohorts, and evaluating clinical applicability to facilitate adoption in decision support systems and precision medicine strategies.

## Electronic supplementary material

Below is the link to the electronic supplementary material.

Supplementary Material 1

Supplementary Material 2

Supplementary Material 3

Supplementary Material 4

Supplementary Material 5

Supplementary Material 6

Supplementary Material 7

Supplementary Material 8

Supplementary Material 9

Supplementary Material 10

Supplementary Material 11

## Data Availability

No datasets were generated or analysed during the current study.
