# Supplementary material for: Comprehensive predictive modeling in subarachnoid hemorrhage: integrating radiomics and clinical variables
Source: Neurosurg Rev. 2025 Jun 24;48(1):528. doi: 10.1007/s10143-025-03679-8 (PMC12187877; doi:10.1007/s10143-025-03679-8)

Supplemental Fig. 3. Model evaluation using white and gray matter for radiomics extraction, stratified by patient age (≥70 years and <70 years). The performance metric shown is the mean AUC of the best model for each fold and seed.

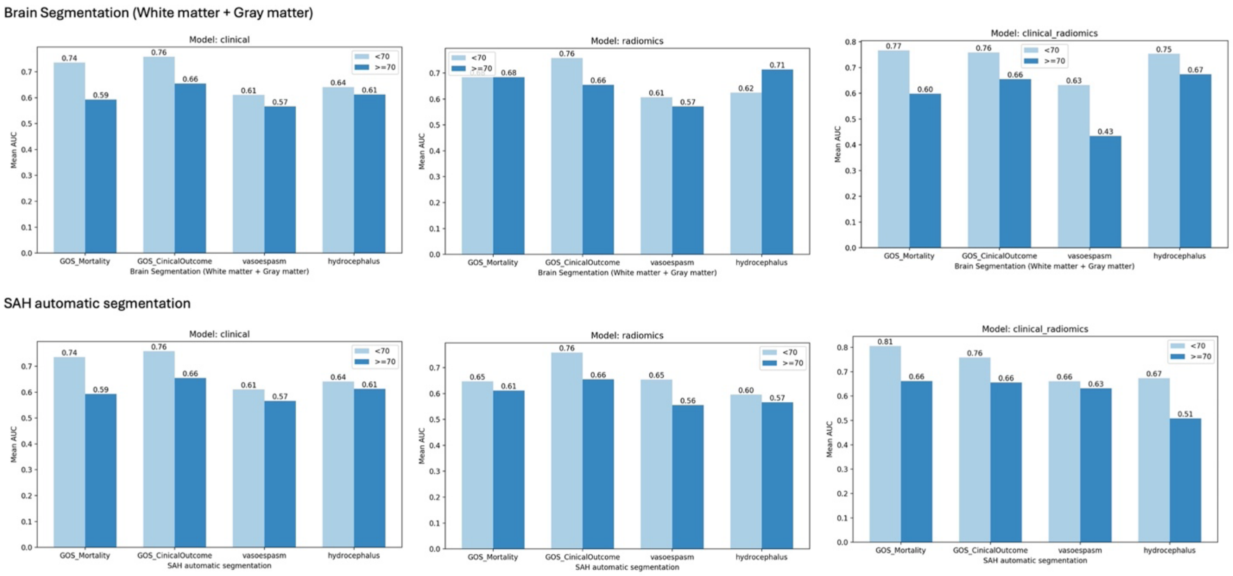

Supplement: Supplementary file 3 — Supplementary Material 3 [file 10143_2025_3679_MOESM3_ESM.pdf]
