# Supplementary material for: Comprehensive predictive modeling in subarachnoid hemorrhage: integrating radiomics and clinical variables
Source: Neurosurg Rev. 2025 Jun 24;48(1):528. doi: 10.1007/s10143-025-03679-8 (PMC12187877; doi:10.1007/s10143-025-03679-8)

Supplemental Fig. 5. Model performance results on the independent test set including the F1-score, accuracy, and confusion matrices for each outcome.

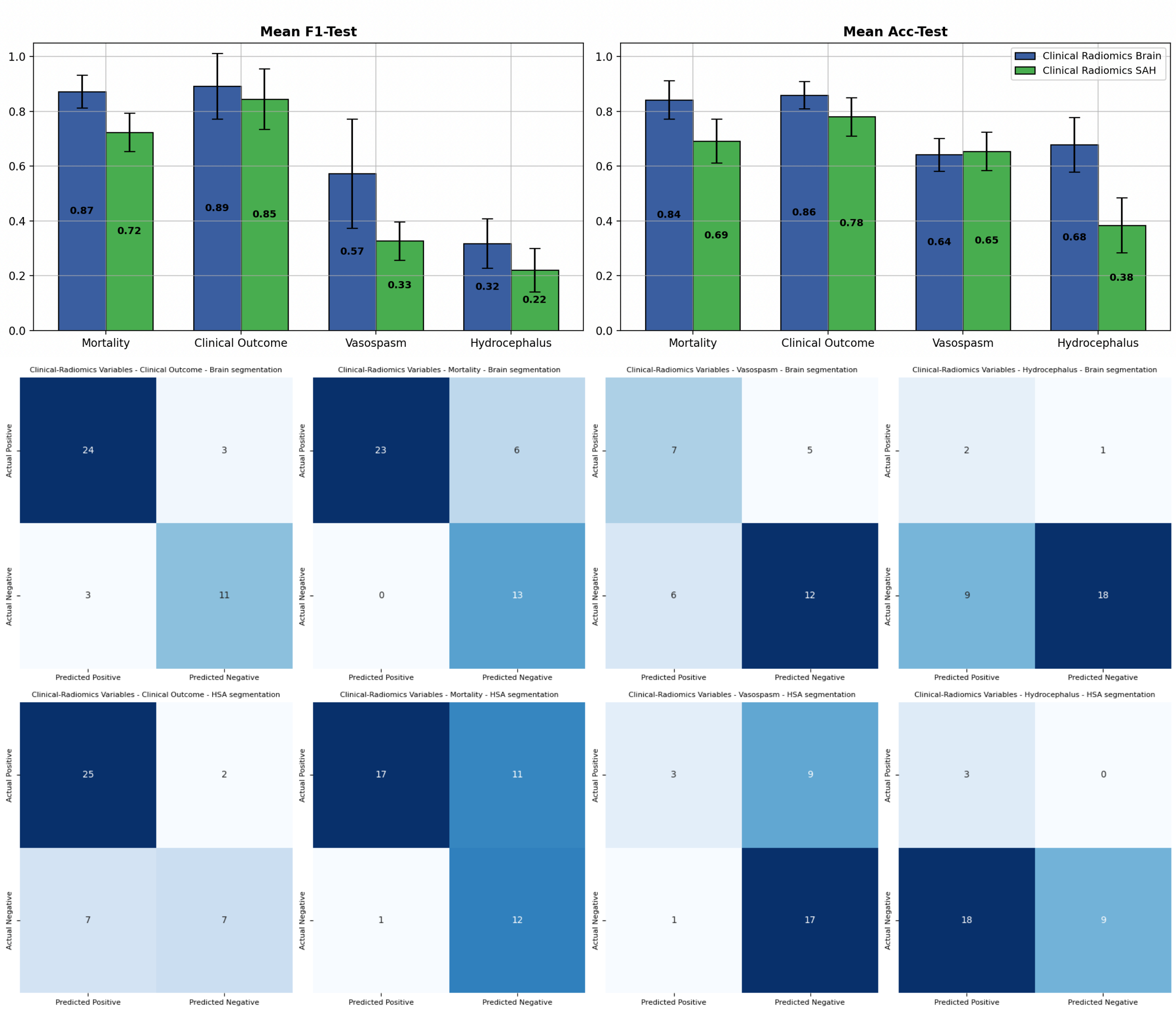

Supplement: Supplementary file 5 — Supplementary Material 5 [file 10143_2025_3679_MOESM5_ESM.pdf]
