# Supplementary material for: Comprehensive predictive modeling in subarachnoid hemorrhage: integrating radiomics and clinical variables
Source: Neurosurg Rev. 2025 Jun 24;48(1):528. doi: 10.1007/s10143-025-03679-8 (PMC12187877; doi:10.1007/s10143-025-03679-8)

Supplemental Fig. 6. SHAP values diagrams from white and grey matter segmentation models for each experiment: using clinical variables, radiomics or combining both.

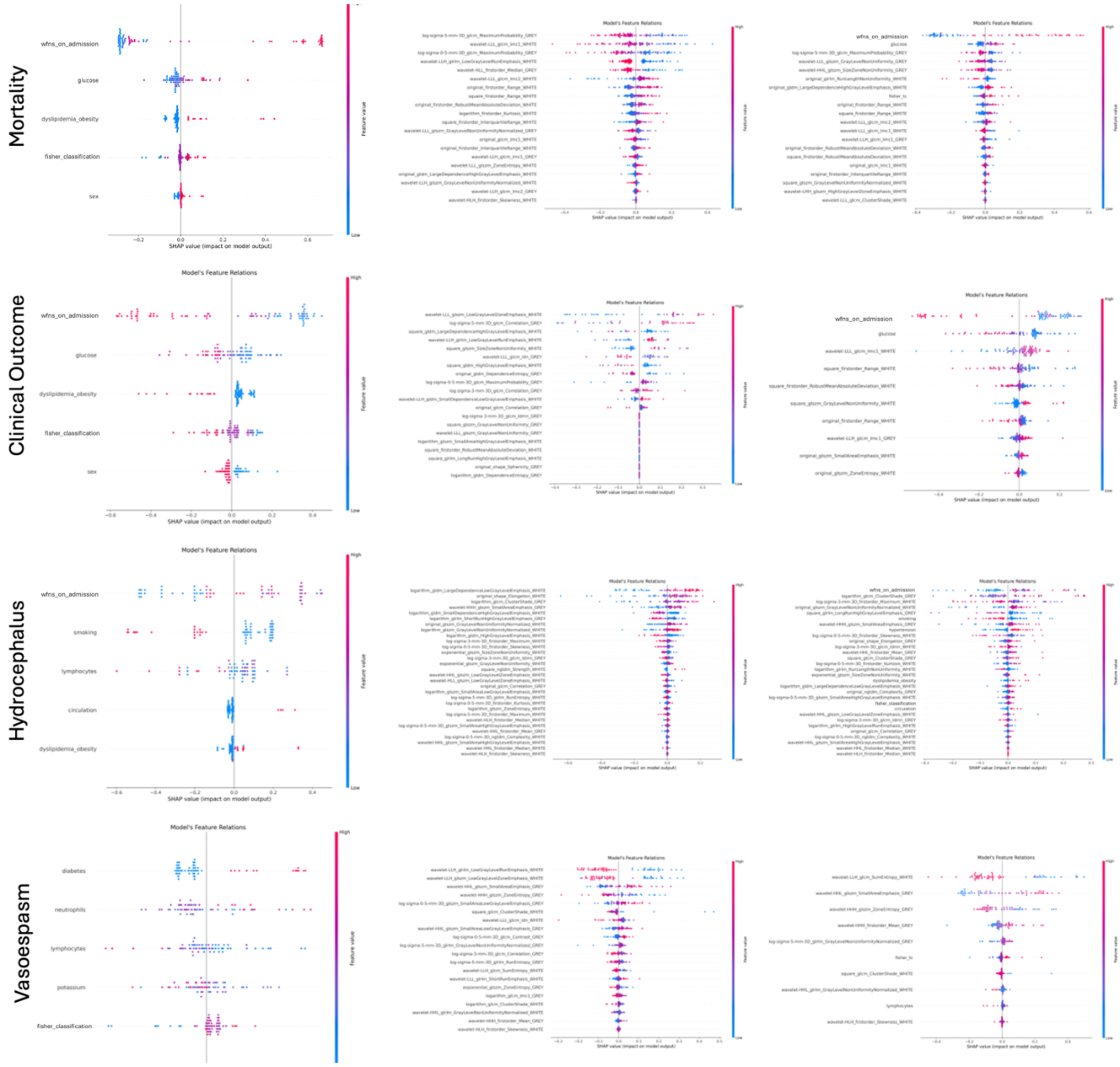

Supplement: Supplementary file 6 — Supplementary Material 6 [file 10143_2025_3679_MOESM6_ESM.pdf]
