# Supplementary material for: Comprehensive predictive modeling in subarachnoid hemorrhage: integrating radiomics and clinical variables
Source: Neurosurg Rev. 2025 Jun 24;48(1):528. doi: 10.1007/s10143-025-03679-8 (PMC12187877; doi:10.1007/s10143-025-03679-8)
Supplement: Supplementary file 7 — Supplementary Material 7 [file 10143_2025_3679_MOESM7_ESM.pdf]

Supplemental Table 1. Definitions and frequencies of comorbidities in the clinical dataset

Comorbidity	Definition	Category	N (%)
**Hypertension**	History of systolic BP ≥130 mmHg, diastolic BP ≥80 mmHg, or antihypertensive treatment at admission.	Yes	41.69%
		No	58.31%
**Smoking**	Active tobacco use at time of admission.	Yes	28.54%
		No	71.46%
**Diabetes Mellitus**	Known diagnosis of diabetes or treatment with insulin/oral hypoglycemics.	Yes	18.60%
		No	81.40%
**Dyslipidemia/Obesity**	History of dyslipidemia (e.g., LDL ≥130 mg/dL) and/or BMI ≥30 kg/m².	Yes	17.86%
		No	82.14%
**Alcoholism**	Documented alcohol use disorder or chronic excessive alcohol intake.	Yes	5.71%
		No	94.29%
**Other drugs**	Active use of recreational drugs (e.g., cocaine, cannabis, opioids).	Yes	3.22%
		No	96.78%
**Family history of SAH**	First-degree relative with history of subarachnoid hemorrhage.	Yes	1.49%
		No	98.51%
**Associated AVM**	Diagnosis of arteriovenous malformation (AVM) associated with aneurysm.	Yes	1.40%
		No	98.60%
