# Supplementary material for: Comprehensive predictive modeling in subarachnoid hemorrhage: integrating radiomics and clinical variables
Source: Neurosurg Rev. 2025 Jun 24;48(1):528. doi: 10.1007/s10143-025-03679-8 (PMC12187877; doi:10.1007/s10143-025-03679-8)
Supplement: Supplementary file 8 — Supplementary Material 8 [file 10143_2025_3679_MOESM8_ESM.pdf]

Supplemental Table 2. Review of automatic HSA segmentation by a neurosurgeon. This table displays five images with significant volume discrepancies between the automatically segmented volumes and the reference volumes. Overestimated segmentations are marked in orange, while underestimated segmentations are indicated in blue.

Case	Volume Reference (mL)	Volume Predicted (mL)	Views (Axial / Coronal)
**Case 277**	15.88	100.3	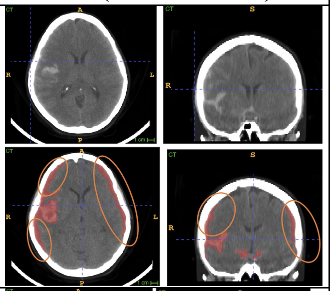
**Case 480**	20.43	101.97	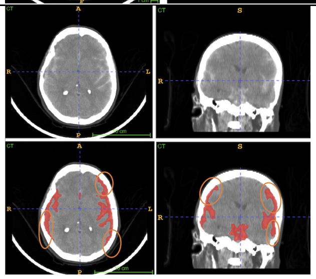
**Case 488**	95.87	19.83	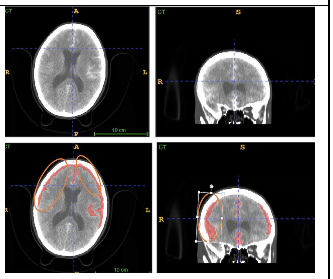
**Case 270**	13.68	87.40	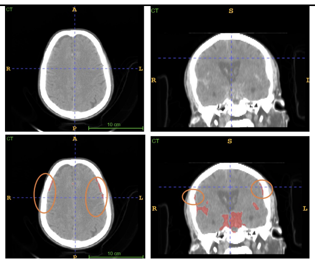
**Case 321**	51.18	124.38	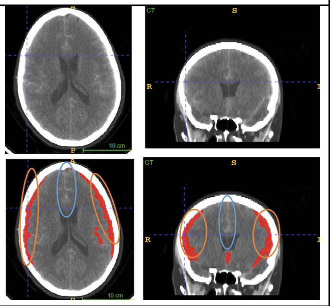
