# Supplementary material for: Comprehensive predictive modeling in subarachnoid hemorrhage: integrating radiomics and clinical variables
Source: Neurosurg Rev. 2025 Jun 24;48(1):528. doi: 10.1007/s10143-025-03679-8 (PMC12187877; doi:10.1007/s10143-025-03679-8)
Supplement: Supplementary file 9 — Supplementary Material 9 [file 10143_2025_3679_MOESM9_ESM.pdf]

Supplemental Table 3. Models evaluation by patient clinical grade. The performance metrics are the mean and standard deviation of the best model for each fold and seed (n=15).

**Experiment**	**Model**	**Group**	**AUC**	**BalAcc**	**Sens**	**Spec**
Clinical	Mortality	Good clinic grade	0.87 ± 0.04	0.77 ± 0.04	0.78 ± 0.04	0.61 ± 0.2
		Poor clinic grade	0.80 ± 0.06	0.74 ± 0.06	0.69 ± 0.06	0.54 ± 0.21
	Clinical outcome	Good clinic grade	0.80 ± 0.09	0.68 ± 0.09	0.26 ± 0.17	0.95 ± 0.04
		Poor clinic grade	0.76 ± 0.05	0.63 ± 0.05	0.72 ± 0.12	0.66 ± 0.21
	Vasoespasm	Good clinic grade	0.70 ± 0.02	0.67 ± 0.02	0.78 ± 0.08	0.69 ± 0.15
		Poor clinic grade	0.64 ± 0.07	0.54 ± 0.07	0.57 ± 0.11	0.56 ± 0.19
	Hydrocephalus	Good clinic grade	0.80 ± 0.07	0.67 ± 0.17	0.73 ± 0.06	0.66 ± 0.30
		Poor clinic grade	0.73 ± 0.08	0.62 ± 0.08	0.65 ± 0.10	0.45 ± 0.20
Radiomics Brain	Mortality	Good clinic grade	0.80 ± 0.03	0.72 ± 0.05	0.75 ± 0.07	0.71 ± 0.2
		Poor clinic grade	0.76 ± 0.05	0.68 ± 0.08	0.67 ± 0.06	0.82 ± 0.21
	Clinical outcome	Good clinic grade	0.80 ± 0.09	0.68 ± 0.09	0.26 ± 0.17	0.95 ± 0.04
		Poor clinic grade	0.76 ± 0.05	0.63 ± 0.05	0.72 ± 0.12	0.66 ± 0.21
	Vasoespasm	Good clinic grade	0.70 ± 0.02	0.67 ± 0.02	0.78 ± 0.08	0.69 ± 0.15
		Poor clinic grade	0.64 ± 0.07	0.54 ± 0.07	0.57 ± 0.11	0.56 ± 0.19
	Hydrocephalus	Good clinic grade	0.80 ± 0.07	0.67 ± 0.17	0.73 ± 0.06	0.66 ± 0.30
		Poor clinic grade	0.73 ± 0.08	0.62 ± 0.08	0.65 ± 0.10	0.45 ± 0.20
Radiomics HSA	Mortality	Good clinic grade	0.70 ± 0.05	0.70 ± 0.05	0.90 ± 0.03	0.50 ± 0.10
		Poor clinic grade	0.65 ± 0.07	0.65 ± 0.07	0.85 ± 0.05	0.45 ± 0.12
	Clinical outcome	Good clinic grade	0.68 ± 0.09	0.68 ± 0.09	0.80 ± 0.04	0.56 ± 0.14
		Poor clinic grade	0.63 ± 0.10	0.63 ± 0.10	0.75 ± 0.06	0.48 ± 0.15
	Vasoespasm	Good clinic grade	0.64 ± 0.03	0.64 ± 0.03	0.76 ± 0.08	0.50 ± 0.12
		Poor clinic grade	0.58 ± 0.06	0.58 ± 0.06	0.70 ± 0.09	0.44 ± 0.10
	Hydrocephalus	Good clinic grade	0.65 ± 0.14	0.65 ± 0.14	0.78 ± 0.07	0.52 ± 0.22
		Poor clinic grade	0.61 ± 0.10	0.61 ± 0.10	0.75 ± 0.08	0.45 ± 0.20
Clinical + Radiomics Brain	Mortality	Good clinic grade	0.65 ± 0.10	0.65 ± 0.10	0.79 ± 0.05	0.55 ± 0.12
		Poor clinic grade	0.63 ± 0.11	0.63 ± 0.11	0.74 ± 0.06	0.50 ± 0.15
	Clinical outcome	Good clinic grade	0.62 ± 0.03	0.62 ± 0.03	0.70 ± 0.08	0.54 ± 0.12
		Poor clinic grade	0.60 ± 0.04	0.60 ± 0.04	0.67 ± 0.09	0.52 ± 0.11
	Vasoespasm	Good clinic grade	0.62 ± 0.08	0.62 ± 0.08	0.76 ± 0.09	0.50 ± 0.10
		Poor clinic grade	0.58 ± 0.06	0.58 ± 0.06	0.71 ± 0.07	0.45 ± 0.09
	Hydrocephalus	Good clinic grade	0.75 ± 0.15	0.75 ± 0.15	0.80 ± 0.06	0.55 ± 0.20
		Poor clinic grade	0.70 ± 0.10	0.70 ± 0.10	0.78 ± 0.08	0.50 ± 0.15
Clinical + Radiomics HSA	Mortality	Good clinic grade	0.68 ± 0.09	0.68 ± 0.09	0.85 ± 0.05	0.52 ± 0.17
		Poor clinic grade	0.66 ± 0.10	0.66 ± 0.10	0.80 ± 0.07	0.55 ± 0.20
	Clinical outcome	Good clinic grade	0.68 ± 0.08	0.68 ± 0.08	0.71 ± 0.12	0.65 ± 0.21
		Poor clinic grade	0.64 ± 0.06	0.64 ± 0.06	0.70 ± 0.10	0.60 ± 0.19
	Vasoespasm	Good clinic grade	0.61 ± 0.01	0.61 ± 0.01	0.78 ± 0.08	0.45 ± 0.14
		Poor clinic grade	0.55 ± 0.05	0.55 ± 0.05	0.72 ± 0.12	0.38 ± 0.15
	Hydrocephalus	Good clinic grade	0.62 ± 0.17	0.62 ± 0.17	0.82 ± 0.04	0.48 ± 0.23
		Poor clinic grade	0.59 ± 0.09	0.59 ± 0.09	0.75 ± 0.11	0.42 ± 0.18
