# Supplementary material for: Comprehensive predictive modeling in subarachnoid hemorrhage: integrating radiomics and clinical variables
Source: Neurosurg Rev. 2025 Jun 24;48(1):528. doi: 10.1007/s10143-025-03679-8 (PMC12187877; doi:10.1007/s10143-025-03679-8)
Supplement: Supplementary file 10 — Supplementary Material 10 [file 10143_2025_3679_MOESM10_ESM.pdf]

Supplemental Table 4. Descriptive statistics of radiological scores used to compute the ictWFNS from test set (n=41)

**Score**	**Mean ± SD**	**Median (IQR)**	**Range**
Hijdra	16.49 ± 8.75	18 (10–24)	0–30
LeRoux	5.44 ± 5.12	4 (1–9)	0–16

Hijdra and LeRoux scores quantify subarachnoid and intraventricular blood burden, respectively. Values are presented as mean ± standard deviation, median (interquartile range), and full range.

**SEBES score**	**Count**	**Percentage (%)**
1	2	5.38%
2	3	7.32%
3	5	12.2%
4	31	75.61%

SEBES is an ordinal 0–4 scale reflecting early brain edema signs on admission CT. Distribution is shown in absolute counts and percentages.
