# Supplementary material for: Comprehensive predictive modeling in subarachnoid hemorrhage: integrating radiomics and clinical variables
Source: Neurosurg Rev. 2025 Jun 24;48(1):528. doi: 10.1007/s10143-025-03679-8 (PMC12187877; doi:10.1007/s10143-025-03679-8)
Supplement: Supplementary file 11 — Supplementary Material 11 [file 10143_2025_3679_MOESM11_ESM.pdf]

Supplemental Table 5. TRIPOD Compliance and Reporting Transparency

This study was conducted and reported in accordance with the TRIPOD (Transparent Reporting of a multivariable prediction model for Individual Prognosis Or Diagnosis) guidelines, ensuring clarity, reproducibility, and methodological transparency in the development and evaluation of the predictive models.

Section/Topic	Item	Checklist Item	Page
**Title and Abstract**	1	Identify the study as developing and/or validating a multivariable prediction model, the target population, and the outcome to be predicted.	2
	2	Provide a summary of objectives, study design, setting, participants, sample size, predictors, outcome, statistical analysis, results, and conclusions.	2
**Introduction**	3a	Explain the medical context (diagnostic or prognostic) and rationale for developing or validating the model, including references to existing models.	3
	3b	Specify the objectives, including whether the study describes development or validation or both.	3
**Methods**	4a	Describe the study design or data source (e.g., cohort, registry), separately for development and validation sets, if applicable.	4
	4b	Specify study dates: start/end of accrual and follow-up.	4
	5a	Describe study setting (e.g., primary/secondary care), number and location of centres.	4
	5b	Describe eligibility criteria for participants.	4
	5c	Give details of treatments received, if relevant.	5
	6a	Define the outcome predicted by the model, including how and when assessed.	5
	6b	Report any actions to blind outcome assessment.	4
	7a	Define all predictors used, including how and when measured.	5
	7b	Report actions to blind predictor assessment.	4
	8	Explain how the sample size was determined.	8
	9	Describe how missing data were handled, including any imputation method.	5
	10a	Describe handling of predictors in the analysis.	5–7
	10b	Specify model type, building procedures (e.g., predictor selection), and internal validation.	7–8
	10d	Specify measures used to assess model performance and compare models.	8
	11	Describe how risk groups were created, if applicable.	8
**Results**	13a	Describe participant flow, number with/without outcome, and follow-up time. A diagram may help.	8–10

	13b	Describe participant characteristics, including missing data.	8–11
	14a	Specify number of participants and outcome events per analysis.	Figure 3
	14b	Report unadjusted associations between predictors and outcome (if done).	N/A - SHAP based interpretation 15-16
	15a	Present full model (all coefficients, intercept, or baseline survival).	11-14
	15b	Explain how to use the model.	18-19
	16	Report model performance measures (with CIs).	12-14 // 17–19
**Discussion**	18	Discuss study limitations (e.g., representativeness, sample size, missing data).	18-19
	19b	Interpret results considering objectives, limitations, and other evidence.	17-19
	20	Discuss clinical use of the model and future research implications.	19
**Other Information**	21	Provide info on supplementary resources (e.g., protocol, calculator, datasets).	20
	22	State funding source and role of funders.	20
